# Duloxetine in the treatment of Major Depressive Disorder: A comparison of efficacy in patients with and without melancholic features

**DOI:** 10.1186/1471-244X-5-1

**Published:** 2005-01-04

**Authors:** Craig H Mallinckrodt, John G Watkin, Chaofeng Liu, Madelaine M Wohlreich, Joel Raskin

**Affiliations:** 1 Lilly Research Laboratories, Eli Lilly and Company, Indianapolis, IN 46285

## Abstract

**Background:**

The most prominent feature of melancholic depression is a near-total loss of the capacity to derive pleasure from activities or other positive stimuli. Additional symptoms can include psychomotor disturbances, anorexia, excessive guilt, and early awakening from sleep. Melancholic patients may exhibit treatment responses and outcomes that differ from those of non-melancholic patients. Pooled data from double-blind, placebo-controlled studies were utilized to compare the efficacy of duloxetine in depressed patients with and without melancholic features.

**Methods:**

Efficacy data were pooled from 8 double-blind, placebo-controlled clinical trials of duloxetine. The presence of melancholic features (DSM-IV criteria) was determined using results from the Mini International Neuropsychiatric Interview (MINI). Patients (aged ≥ 18 years) meeting DSM-IV criteria for major depressive disorder (MDD) received duloxetine (40–120 mg/d; melancholic, N = 759; non-melancholic, N = 379) or placebo (melancholic, N = 519; non-melancholic, N = 256) for up to 9 weeks. Efficacy measures included the 17-item Hamilton Rating Scale for Depression (HAMD_17_) total score, HAMD_17 _subscales (Maier, anxiety, retardation, sleep), the Clinical Global Impression of Severity (CGI-S) and Patient Global Impression of Improvement (PGI-I) scales, and Visual Analog Scales (VAS) for pain.

**Results:**

In data from all 8 studies, duloxetine's advantage over placebo did not differ significantly between melancholic and non-melancholic patients (treatment-by-melancholic status interactions were not statistically significant). Duloxetine demonstrated significantly greater improvement in depressive symptom severity, compared with placebo, within both melancholic and non-melancholic cohorts (p ≤ .001 for HAMD_17 _total score, CGI-S and PGI-I). When analyzed by gender, the magnitude of improvement in efficacy outcomes did not differ significantly between duloxetine-treated male and female melancholic patients. In the two studies that assessed duloxetine 60 mg once-daily dosing, duloxetine-treated melancholic patients had significantly greater improvement compared with placebo on HAMD_17 _total score, CGI-S, PGI-I, 3 of 4 subscales of the HAMD_17_, and VAS overall pain severity (p < .01). Estimated probabilities of response and remission were significantly greater for melancholic patients receiving duloxetine 60 mg QD compared with placebo (response 74.7% vs. 42.2%, respectively, p < .001; remission 44.4% vs. 24.7%, respectively, p = .002

**Conclusions:**

In this analysis of pooled data, the efficacy of duloxetine in patients with melancholic features did not differ significantly from that observed in non-melancholic patients.

## Background

Although the first appearance of the term "melancholia" dates back to antiquity[1], its use was in decline until it was re-adopted in 1980 by the authors of the *Diagnostic and Statistical Manual of Mental Disorders, 3*^*rd*^*Edition *(DSM-III) to describe a subtype of major depressive disorder (MDD). Although revised in DSM-III-R, the current DSM-IV "With Melancholic Features Specifier" represents a return to the older DSM-III definition. According to DSM-IV criteria, the principal diagnostic feature exhibited by patients with melancholic depression is a loss of pleasure in all, or almost all, activities or a lack of reactivity to usually pleasurable stimuli [2]. Additional symptoms include diurnal variation (depression worse in the morning), psychomotor disturbances, anorexia, excessive guilt, and early awakening from sleep. Estimates of the prevalence of melancholic features among patients diagnosed with MDD range from 16% to 53% [3-6], although the prevalence may be as high as 76% among inpatients [6].

Melancholia is encountered equally in both genders [7], but is observed more frequently in older patients [4,8]. While melancholia is associated with an increased comorbidity of anxiety disorders, panic disorder, and phobia [9], melancholic patients have a significantly lower suicide rate than non-melancholics [10].

A considerable body of evidence suggests that biological differences exist between melancholic and non-melancholic depression. The hypercortisolism of melancholia has been described as perhaps the best documented finding in biological psychiatry[11]. Many of the features exhibited by melancholic patients closely resemble those that occur reflexively in non-depressed populations during acutely stressful or threatening situations [12]. Thus, depressed patients with melancholic features consistently demonstrate an activation of the hypothalamic-pituitary-adrenocortical (HPA) axis[13], resulting in laboratory findings of dexamethasone non-suppression. Melancholic patients also have an activated corticotrophin-releasing hormone (CRH) system and may have diminished activities of the growth hormone and reproductive axes[14]. When compared with non-melancholic depressed patients, melancholics have also been shown to exhibit lower concentration of nighttime serum melatonin [15], lower plasma serotonin (5-HT) concentrations [16,17], and an impaired *in vivo *immune response [18,19].

Given the distinct clinical and physiological features associated with melancholia, it is perhaps not surprising that melancholic patients may exhibit treatment responses and outcomes that differ from those of non-melancholic depressed patients. In reviewing a number of long-term studies, Parker *et al *concluded that melancholics appear to respond well to treatment of individual episodes but have frequent recurrences, while non-melancholics may respond less rapidly and less completely in any individual episode but show a general pattern of improvement over time [20]. Overall, melancholics appear to have higher rehospitalization rates and greater illness morbidity over an extended period [20].

Melancholic patients are less likely to respond to non-specific supportive therapies, such as cognitive behavior therapy or interpersonal psychotherapy, and more often require pharmacotherapy to achieve a successful treatment outcome [21,22]. Within the context of clinical trials, this apparent lack of response to non-specific therapies is frequently manifested in the form of a markedly lower placebo response rate among melancholic, when compared with non-melancholic, patients [23-27]. Thus, in some studies, antidepressant-treated melancholic patients have demonstrated significantly greater improvements compared with placebo while a non-melancholic cohort has failed to achieve separation from placebo. However, the drug response is usually of similar magnitude in all patients, and the difference in outcome can be traced to significantly different placebo responses within melancholic and non-melancholic patients [26,28].

Although a number of studies have investigated the relative efficacy of specific classes of antidepressant medications in melancholic patients, results have been somewhat inconsistent [26]. While tricyclic antidepressants (TCAs) have demonstrated superiority over selective serotonin reuptake inhibitors (SSRIs) in some studies of melancholic depression [29-32], results from other head-to-head studies have failed to support these findings [33-35]. Thus, the current consensus is that SSRIs and TCAs demonstrate comparable efficacy for the treatment of melancholic depression [13,36]. With regard to safety and tolerability, however, SSRIs offer considerable advantages since they lack the anticholinergic, antihistaminergic, and cardiotoxic effects associated with TCAs [37]. As a result, SSRIs are now recognized as the first-line treatment for melancholia.

The antidepressant duloxetine is a balanced and potent dual reuptake inhibitor of 5-HT and norepinephrine (NE). The efficacy of duloxetine in the treatment of MDD has been established in randomized, double-blind, placebo-controlled studies of up to 9 weeks duration [38]. In the present study, pooled data from 8 clinical trials of duloxetine were utilized to compare treatment outcomes in melancholic patients with those in non-melancholic patients.

## Methods

### Study Design

All 8 studies included in these analyses were randomized, multicenter, double-blind, placebo-controlled clinical trials and represented all of the double-blind studies included in the New Drug Application reviewed by regulatory agencies for duloxetine's indication in MDD. All studies incorporated double-blind, variable-expected duration placebo lead-in periods to blind patients and investigators to the start of active therapy. Six studies also utilized an active comparator – fluoxetine (20 mg QD) in Studies **1 **and **2**, and paroxetine (20 mg QD) in Studies **3**, **4**, **7**, and **8**. Study protocols were reviewed and approved by the ethical review board at each center, in accordance with the principles of the Declaration of Helsinki, and all patients provided written informed consent prior to the administration of any study procedures or study drug. The numbers of patients randomized in each study are summarized in Table 1. Detailed safety and efficacy results from Studies **1 **[39], **4 **[40], **5 **[41], and **6 **[42] have been published previously.

**Table 1:** Numbers of randomized patients^‡^

**Study**	**Placebo**	**SSRI^a^**	**Duloxetine**			
			
			**40 mg/d^b^**	**60 mg QD**	**80 mg/d^c^**	**120 mg/d^d^**
**1**	70 (80.0)	33 (72.7)	-	-	-	70 (77.1)
**2**	74 (73.0)	37 (86.5)	-	-	-	82 (69.5)
**3**	90 (55.6)	89 (64.0)	91 (62.6)	-	84 (60.7)	-
**4**	89 (66.3)	87 (63.2)	86 (72.1)	-	91 (69.2)	-
**5**	121 (69.4)	-	-	122 (63.1)	-	-
**6**	139 (62.6)	-	-	128 (62.5)	-	-
**7**	93 (66.7)	86 (68.6)	-	-	95 (65.3)	93 (66.7)
**8**	99 (67.7)	97 (69.1)	-	-	93 (71.0)	103 (66.0)
**TOTAL**	775 (67.0)	429 (68.5)	1138 (66.7)			

^‡ ^Data presented in the form T (% M), where T = total number of patients, % M = percentage of patients with melancholic features

a. SSRI in Studies 1 and 2 is fluoxetine (20 mg QD). SSRI in Studies 3, 4, 7, and 8 is paroxetine (20 mg QD).

b. Administered 20 mg twice-daily (BID)

c. Administered 40 mg BID

d. Administered 60 mg BID. Studies **1 **and **2 **incorporated a forced titration from 20 mg BID to 60 mg BID.

### Patients

In all studies, patients were 18 years of age or older and met criteria for MDD as defined by the *Diagnostic and Statistical Manual of Mental Disorders, 4*^*th*^*Edition *(DSM-IV) [43]. The diagnosis of MDD was confirmed by the Mini International Neuropsychiatric Interview (MINI) [44], a standardized diagnostic interview based on DSM-IV criteria. Patients had a 17-item Hamilton Rating Scale for Depression (HAMD_17_)[45] total score ≥ 15 and a Clinical Global Impression of Severity (CGI-S)[46] score ≥ 4 at the screening and second study visits. The presence of melancholic features (DSM-IV criteria) was determined using results from the MINI:

"*Either feature 1 or 2 in Criteria A AND three (or more) features from Criteria B must be present to qualify for melancholic features.*

A. Either of the following, occurring during the most severe period of the current episode.

1. Loss of pleasure in all, or almost all, activities;

2. Lack of reactivity to usually pleasurable stimuli

B. Three (or more) of the following:

1. Distinct quality of depressed mood;

2. Depression regularly worse in the morning;

3. Early morning awakening (at least 2 hours before usual time of awakening);

4. Marked psychomotor retardation or agitation;

5. Significant anorexia or weight loss;

*6. Excessive or inappropriate guilt.*"

Patients were excluded for the following reasons: a current and primary Axis I disorder, other than MDD; an Axis II disorder which could interfere with protocol compliance; lack of response of the current depressive episode to two or more adequate courses of antidepressant therapy; serious medical illness; a serious risk of suicide; a history of substance abuse or dependence within the last year, or a positive urine drug screen.

Concomitant medications with primarily central nervous system activity were not permitted, with the exception of episodic use of chloral hydrate or zolpidem for insomnia. Chronic use of prescription analgesic medications was not allowed; episodic use was permitted at the discretion of the physician in charge of the study. Use of anti-hypertensive medications was not permitted unless the patient had been on a stable dose for at least 3 months prior to study entry.

### Data Pooling Strategies

Efficacy analyses were performed on three sets of data, obtained using the following pooling strategies:

(A) "All Studies" – data from all 8 studies were pooled. Placebo: melancholic (n = 519; 67.0%), non-melancholic (n = 256; 33.0%). Duloxetine (40–120 mg/d): melancholic (n = 759; 66.7%), non-melancholic (n = 379; 33.3%). Duloxetine was compared with placebo in one set of analyses. In a second set of analyses using data from the 6 SSRI-controlled studies, duloxetine was compared with fluoxetine and paroxetine: Placebo: melancholic (n = 348; 67.6%), non-melancholic (n = 167; 32.4%). Duloxetine (40–120 mg/d): melancholic (n = 602; 67.8%), non-melancholic (n = 286; 32.2%). SSRI: melancholic (n = 294; 68.5%), non-melancholic (n = 135; 31.5%);

(B) "Positive Studies" – data from placebo- and duloxetine-treated patients were pooled from the 6 studies (**1**, **4**, **5**, **6**,**7**, and **8**) that demonstrated a significant advantage for duloxetine over placebo on the primary efficacy measure. Placebo: melancholic (n = 415; 67.9%), non-melancholic (n = 196; 32.1%). Duloxetine (40–120 mg/d): melancholic (n = 594; 67.4%), non-melancholic (n = 287; 32.6%);

(C) "Focus Studies" – data were pooled from the 2 studies (**5 **and **6**) that compared duloxetine 60 mg once-daily with placebo. Placebo: melancholic (n = 171; 65.8%), non-melancholic (n = 89; 34.2%). Duloxetine (60 mg/d): melancholic (n = 157; 62.8%), non-melancholic (n = 93; 37.2%).

Strategy A facilitated assessments of differential efficacy in the largest possible data set. While the inclusion of all available data has obvious advantages, the presence of failed studies could mask differential treatment effects. If a study failed to detect an overall effect it is unlikely to help detect differential subgroup effects. Therefore strategy B essentially served as a robustness check for strategy A. Pooling strategy C facilitated assessments at the recommended target dose.

### Efficacy Measures

In all 8 studies, the primary efficacy outcome was mean change from baseline to endpoint in HAMD_17 _total score. Other efficacy measures assessed in all studies included HAMD_17 _subscales: anxiety/somatization (Items 10, 11, 12, 13, 15, and 17), Maier (Items 1, 2, 7, 8, 9, and 10), retardation (Items 1, 7, 8, and 14), and sleep (Items 4, 5, and 6); the CGI-S scale; and the Patient Global Impression of Improvement (PGI-I) scale [46]. Response was defined as a decrease from baseline of at least 50% on the HAMD_17 _total score. Remission was defined as a HAMD_17 _total score ≤ 7. In Studies **3 **– **8**, the severity of painful physical symptoms was assessed using Visual Analog Scales (VAS) for pain [47].

### Statistical analyses

Patients with missing melancholia status were not included in the analyses. All other patients with a baseline and at least one postbaseline observation were included in the analyses. Mean changes from baseline to last observation (carried forward) in HAMD_17 _total score, CGI-S, and PGI-I were assessed using an analysis of covariance (ANCOVA) with models that included baseline score, treatment, melancholia status (features present Yes/No), investigator, and the treatment-by-melancholia status interaction as independent variables. Hereafter this analysis is referred to as LOCF mean change.

The treatment-by-melancholic status interaction was the main basis upon which differential treatment effects between the subgroups were assessed. Contrasts between duloxetine and placebo within the melancholic and non-melancholic subgroups were used to assess the clinical relevance of treatment effects.

Longitudinal mean changes and categorical changes (estimated probabilities) were assessed using a likelihood-based mixed-effects model repeated measures (MMRM) approach. Models for mean changes included treatment, visit, investigator, baseline HAMD_17 _value, melancholia status, and the two-way and three-way interactions between treatment, visit, and melancholia status. Hereafter this analysis is referred to as MMRM mean change. The categorical longitudinal analyses were similar in concept to the longitudinal mean change analyses, but simplifications were necessary to reduce the computational complexity.

The categorical analyses were applied only to patients with melancholic features so that the main effect of melancholic status and its two-way and three-way interactions with visit and treatment could be deleted. Therefore, the model for the categorical analyses included treatment, visit, investigator, baseline HAMD_17 _value, and the treatment-by-visit interaction. A logit link function and a binomial error structure were included to account for the non-linearity of the response and the non-normality of the data, respectively. Hereafter, this analysis is referred to as categorical MMRM. Remission and response rates at last observation were assessed using Fisher's Exact test.

The LOCF mean change analysis of HAMD_17_, CGI-S and PGI-I was applied to all three databases. The focus for the analyses was on all studies and the positive studies with the primary aim of detecting differential effects of duloxetine in patients with and without melancholic features. The MMRM mean change and categorical MMRM analyses were then applied to a wide variety of outcomes from the focus studies in order to gain an in-depth perspective on the efficacy of duloxetine in patients with melancholic features. In addition, LOCF analyses of data from the focus studies were conducted in order to assess robustness of the MMRM results.

## Results

### Patient characteristics

Baseline patient demographics are summarized in Table 2. The prevalence of melancholic features in the overall patient population was 67.1% (1572/2342). The melancholic cohort had a significantly higher proportion of females compared with the non-melancholic group (69.5% vs. 60.8%, p < .001). Melancholic patients also had a significantly lower mean body weight than non-melancholics (77.7 kg vs. 81.3 kg, p < .001). Mean age at enrollment did not differ significantly between melancholic and non-melancholic patients.

Mean baseline HAMD_17 _total scores and CGI-S scores were significantly higher (more severe illness) in melancholic patients compared with non-melancholics (HAMD_17_: 22.3 vs. 20.5, p < .001; CGI-S: 4.41 vs. 4.26, p < .001). There was a marginally significant difference in VAS overall pain severity at baseline (31.7 vs. 31.0 for melancholic and non-melancholic groups, respectively; p = .053), although the clinical relevance of this small difference is questionable.

**Table 2:** Baseline demographics and illness severity (all studies)^†^

	**Melancholic (N = 1572)**	**Non-melancholic (N = 770)**	**p-value**
**Gender**, n (%)			
Female	1092 (69.5)	468 (60.8)	<.001
**Age**, y			
Mean (SD)	42.1 (12.2)	43.4 (12.8)	.347
**Age range**			
Min – Max	18 – 82	18 – 82	-
**Weight**, kg			
Mean (SD)	77.7 (20.5)	81.3 (21.4)	<.001
**HAMD_17 _Total Score**			
Mean (SD)	22.3 (3.9)	20.5 (3.2)	<.001
**CGI-Severity**			
Mean (SD)	4.41 (0.56)	4.26 (0.48)	<.001
**VAS Overall Pain**			
Mean (SD)	31.7 (25.3)	31.0 (26.0)	.053

† Includes patients from paroxetine and fluoxetine treatment arms (N = 429).

### Efficacy – All studies and positive studies

Analyses of mean changes from baseline to last observation (LOCF mean change) from all eight studies and the six positive studies are summarized in Table 3. In both melancholic and non-melancholic patients, duloxetine demonstrated significant advantages over placebo in HAMD_17 _total score, CGI-S and PGI-I (p ≤ .001). Treatment-by-melancholic status interactions were not significant for HAMD_17 _total score, CGI-S, or PGI-I, in either data set (p > .50 for each comparison). Using pooled data from all studies, the effect size for HAMD_17 _total score was 0.33 in melancholic patients and 0.32 in non-melancholics. In the six positive studies, the effect size for HAMD_17 _total score was 0.39 in melancholic patients compared with 0.43 in non-melancholics.

**Table 3:** Mean changes from baseline to last observation in all studies and the subset of positive studies

	**Melancholic Status**	**Mean Change (SD)**		**p-value^a^**	**p-value^b^**
				
		**Duloxetine**	**Placebo**		
**HAMD_17 _Total Score**					
**All Studies**	MEL (n = 1249)	-8.97 (7.36)	-6.57 (7.24)	<.001	
	NON-MEL (n = 621)	-8.25 (6.55)	-6.20 (6.22)	<.001	.651
**Positive Studies**	MEL (n = 984)	-9.83 (7.20)	-7.03 (7.06)	<.001	
	NON-MEL (n = 474)	-9.15 (6.23)	-6.47 (6.27)	<.001	.781
**CGI-Severity**					

**All Studies**	MEL (n = 1251)	-1.52 (1.25)	-1.11 (1.21)	<.001	
	NON-MEL (n = 621)	-1.47 (1.23)	-1.14 (1.19)	.001	.511
**Positive Studies**	MEL (n = 986)	-1.62 (1.25)	-1.16 (1.20)	<.001	
	NON-MEL (n = 474)	-1.54 (1.13)	-1.15 (1.17)	<.001	.679
**PGI-Improvement**^†^	**Mean (SD)**				

**All Studies**	MEL (n = 1249)	2.67 (1.27)	3.07 (1.27)	<.001	
	NON-MEL (n = 621)	2.71 (1.31)	3.17 (1.27)	<.001	.865
**Positive Studies**	MEL (n = 984)	2.64 (1.26)	3.09 (1.27)	<.001	
	NON-MEL (n = 474)	2.70 (1.28)	3.18 (1.32)	<.001	.912

a. p-value for duloxetine vs. placebo

b. p-value for treatment-by-melancholia status interaction

† Mean score (SD). Lower mean score indicates greater improvement.

Within the subset of melancholic patients, treatment-by-gender interactions for mean change in HAMD_17 _total score, mean change in CGI-S score, and mean endpoint PGI-I score were not statistically significant, indicating that duloxetine's efficacy was similar in male and female melancholic patients (Table 4). In pooled data from all studies, the effect size for HAMD_17 _total score was 0.29 in male melancholic patients compared with 0.35 in female melancholics. Effect sizes for CGI-S score were 0.35 vs. 0.33 for male and female melancholic patients, respectively, while PGI-I effect sizes were 0.26 (males) vs. 0.34 (females).

**Table 4:** Mean changes from baseline to last observation by gender in melancholic patients

	**Gender**	**Mean Change (SD)**		**p-value^a^**	**p-value^b^**
				
		**Duloxetine**	**Placebo**		
**HAMD_17 _Total Score**	Male (n = 386)	-7.99 (7.04)	-5.95 (7.13)	.003	.932
	Female (n = 863)	-9.42 (7.46)	-6.83 (7.29)	<.001	
**CGI-Severity**	Male (n = 386)	-1.41 (1.28)	-0.97 (1.22)	.001	.668
	Female (n = 865)	-1.57 (1.24)	-1.17 (1.20)	<.001	
	**Mean (SD)**				
**PGI-Improvement^†^**	Male (n = 385)	2.80 (1.27)	3.13 (1.23)	.009	.877
	Female (n = 864)	2.61 (1.26)	3.04 (1.29)	<.001	

a. p-value for duloxetine vs. placebo

b. p-value for treatment-by-gender interaction

† Mean score (SD). Lower mean score indicates greater improvement.

In melancholic patients, treatment-by-age interactions for mean change in HAMD_17 _total score were not significant at thresholds of age 55 (p = .777), age 60 (p = .387), or age 65 (p = .540), indicating that the efficacy of duloxetine did not differ between older and younger melancholic patients irrespective of the old/young age threshold.

In the 6 studies that included an SSRI comparator, there were no significant differences in baseline-to-endpoint changes in efficacy measures between duloxetine and SSRI treatment groups (Table 5). In addition, treatment-by-therapy interactions for mean change in HAMD_17 _total score, mean change in CGI-S score, and mean endpoint PGI-I score were not statistically significant, indicating that the relative efficacy of duloxetine and SSRIs did not differ significantly between melancholic and non-melancholic patients (Table 5).

**Table 5:** Mean changes from baseline to last observation in active comparator studies

	**Melancholic Status**	**Mean Change (SD)**		**p-value^a^**	**p-value^b^**
				
		**Duloxetine**	**SSRI**		
**HAMD_17 _Total Score**	MEL (n = 878)	-8.85 (7.39)	-8.60 (7.68)	.709	.784
	NON-MEL (n = 416)	-8.18 (6.52)	-7.59 (6.70)	.897	
**CGI-Severity**	MEL (n = 880)	-1.53 (1.26)	-1.52 (1.33)	.979	.865
	NON-MEL (n = 416)	-1.46 (1.24)	-1.40 (1.24)	.474	
	**Mean (SD)**				

**PGI-Improvement^†^**	MEL (n = 877)	2.67 (1.28)	2.64 (1.34)	.484	.817
	NON-MEL (n = 416)	2.64 (1.29)	2.57 (1.02)	.077	

a. p-value for duloxetine vs. SSRI

b. p-value for treatment-by-melancholia status interaction

† Mean score (SD). Lower mean score indicates greater improvement.

### Efficacy – focus studies

**Figure 1:**
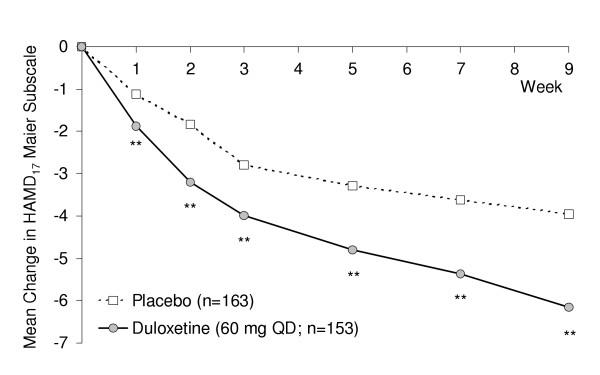
Mean changes in HAMD_17 _Maier subscale for melancholic patients receiving duloxetine (60 mg QD) or placebo. ** p < .005 vs. placebo.

Analyses of mean changes from baseline to Week 9 (MMRM mean change) for melancholic patients in the two focus studies (Studies **5 **and **6**) are summarized in Table 6. Melancholic patients receiving duloxetine had significantly greater improvement in mean HAMD_17 _total score and HAMD_17 _Maier subscale compared with those receiving placebo (p < .001). Significant differences from placebo first occurred at Week 1 (Maier subscale, Figure 1) or Week 2 (total score) and were present at all subsequent visits. Significant advantages for duloxetine over placebo for mean changes to Week 9 among melancholic patients were also observed on the HAMD_17 _retardation and sleep subscales, but not for the anxiety subscale (p = .230). On both clinician-rated (CGI-S) and patient-rated (PGI-I) assessments of global improvement, duloxetine-treated melancholic patients had significantly greater mean improvements compared with melancholics receiving placebo (p < .001). Robustness of the MMRM results was confirmed in that significant differences were also observed in LOCF mean change analyses.

**Table 6:** Mean changes from baseline to week 9 in melancholic patients (focus studies)

	**Mean Change (SE)**		**p-value**
		
	**Duloxetine 60 mg QD (n = 153)**	**Placebo (n = 163)**	
**HAMD_17 _Total Score**	-11.02 (0.65)	-7.51 (0.61)	<.001
**Subscales**			
Maier	-6.16 (0.36)	-3.95 (0.34)	<.001
Anxiety	-2.65 (0.22)	-2.29 (0.21)	.230
Retardation	-4.52 (0.26)	-2.67 (0.24)	<.001
Sleep	-1.78 (0.17)	-1.17 (0.16)	.007
**VAS Overall Pain^a^**	-6.12 (-22.8)	0.13 (0.48)	.002
**CGI-Severity**	-1.84 (0.11)	-1.30 (0.10)	<.001
**PGI-Improvement^b^**	2.47 (0.11)	3.08 (0.11)	<.001

CGI = Clinical Global Impression; HAMD = Hamilton Rating Scale for Depression; PGI = Patient Global Impression

a. Values are mean change (percentage mean change), and p value is from main effect of treatment

b. Mean score (SE). Lower mean score indicates greater improvement.

**Figure 2:**
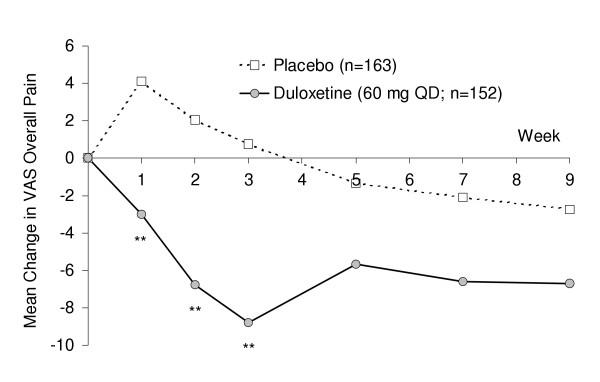
Mean changes in VAS overall pain severity for melancholic patients receiving duloxetine (60 mg QD) or placebo. ** p < .005 vs. placebo.

Mean changes from baseline (MMRM mean change) for VAS overall pain were also assessed. Figure 2 shows a visitwise plot of mean changes in VAS overall pain severity for melancholic patients. For the main effect of treatment (pooling results from all visits – interpreted similar to an area under the curve analysis) duloxetine-treated melancholic patients had significantly greater improvement compared with placebo. Duloxetine's advantage over placebo in treating the painful physical symptoms did not vary substantially between patients with and without melancholic features. However, the response profiles were somewhat different in that response to placebo was generally lower in patients with melancholic features compared with non-melancholic patients. For example, the mean percentage improvement in overall pain among non-melancholic patients receiving placebo was 15.6%, compared with a 0.5% worsening in overall pain among placebo-treated melancholic patients.

Estimated probabilities (categorical MMRM analyses) of remission at Week 9 were significantly higher for melancholic patients treated with duloxetine (60 mg QD) compared with placebo (44.4% vs. 24.7%, respectively; p = .002). The estimated probability of response among melancholic patients was 74.7% for duloxetine compared with 42.2% for placebo (p < .001). The advantage of duloxetine over placebo in remission and response rates was also significant in LOCF analyses (p = .032 and p = .002, respectively).

## Discussion

The magnitude of duloxetine's advantage over placebo was generally similar in melancholic and non-melancholic patients. Treatment-by-melancholic status interactions were not significant for any of the three assessed efficacy measures (HAMD_17 _total score, CGI-S, PGI-I). These results suggest that duloxetine's efficacy does not differ substantially between melancholic and non-melancholic patients. Furthermore, within the group of melancholic patients, duloxetine's treatment effects were similar in male and female patients (treatment-by-gender interactions were not statistically significant). One exception was noted in the anxiety subscale of the HAMD_17_. In the two focus studies, duloxetine did not achieve separation from placebo in patients with melancholic features, but demonstrated efficacy in non-melancholic patients. The reason for the disparity in outcomes between melancholic and non-melancholic patients on the HAMD_17 _anxiety subscale is unclear.

In previous studies, the prevalence of melancholic features in populations of depressed outpatients has ranged from 16% to 53%. The substantial variation in the reported rates of melancholia may be a result of the numerous criteria used to define melancholic features over the past two decades, including DSM-III, DSM-III-R, DSM-IV, and the Newcastle 1 Depression Rating Scale  (N1) [48]. In the present study of depressed outpatients, the prevalence of melancholic features was substantially higher (67.1%) than previous estimates, and merits further comment. At baseline screening all patients were required to meet DSM-IV criteria for MDD, while the presence of melancholic features (DSM-IV criteria) was determined using results from the MINI (further details are provided in the Methods section). The high prevalence of melancholic features was consistent across all 8 clinical trials regardless of geographic location (two studies were conducted in Eastern Europe, six were conducted in the United States). Given the large number of investigative sites (over 140 sites across the 8 studies), and the fact that melancholic features were not used as an inclusion or exclusion criterion in any of the studies, it is unlikely that raters were artificially inflating the number of patients classified as melancholic. A more likely explanation appears to be that the screening method (*i.e. *the MINI plus DSM-IV criteria) produced a substantial number of false positive results with regard to melancholic features. It is possible that the use of an alternative melancholia screening tool, such as the CORE scale developed by Parker et al [49], would have identified a smaller and more well-defined group of melancholic patients. Thus, in a study evaluating the CORE measure of melancholia against the DSM-IV construct, the CORE criteria identified patients with neuroendocrine disturbance whereas DSM-IV criteria did not [50]. In a wider context, the fact that such a high proportion of patients in the current study met DSM-IV criteria for melancholia may raise questions regarding the validity of melancholia as a separate clinical entity [50], and whether it should be considered simply as a variant of MDD which differs only in severity [51,52]. Although the current results cannot directly address concerns as to the validity of melancholia, the unusually high proportion of melancholic patients identified at baseline suggests that the use of the MINI together with DSM-IV criteria may well be an inadequate screening tool for such purposes.

There was no significant difference in age between melancholic and non-melancholic patient groups, despite the fact that melancholic features are more common in older patients. Patients with melancholic features had significantly lower mean body weight at baseline compared to non-melancholics, with a between-group difference of 3.6 kg (8.0 lbs). Since one of the DSM-IV criteria for melancholic features is "significant anorexia or weight loss", this result does not appear surprising. However, given that the melancholic cohort contained a significantly greater proportion of females when compared with the non-melancholic group, the lower body weight among melancholics may be an artifact of gender rather than of MDD subtype. For example, in a recent study of 1694 depressed patients, Berlin *et al *reported that the presence of melancholic features was not associated with lower body mass index [53].

As expected, melancholic patients exhibited a greater severity of illness at baseline compared with non-melancholics, with a mean baseline HAMD_17 _score almost two points higher (approximately one-half standard deviation) in the melancholic group. Melancholic patients also had significantly higher baseline CGI-S scores when compared to those without melancholic features. Although melancholic patients had a slightly higher overall pain severity at baseline (as assessed using the self-rated VAS measure of overall pain), the difference only approached statistical significance and its clinical relevance is questionable.

A frequent finding in placebo-controlled antidepressant studies is that placebo responses among melancholic patients are smaller than those observed in non-melancholic patients [23-25,54], although this is not always the case [55]. In the present assessment, mean changes in HAMD_17_, CGI-S or PGI-I scores among both placebo- and duloxetine-treated melancholic patients were slightly larger than those of non-melancholic patients, resulting in a very similar drug-placebo difference in both patient groups. In measures of pain severity there was evidence of greater placebo response among non-melancholic patients; however, duloxetine's advantage over placebo was similar in melancholic and non-melancholic patients, since patients without melancholic features exhibited more robust responses to both drug *and *placebo, when compared with melancholic patients.

There does not appear to be a clear consensus as to which class of antidepressant medication is most efficacious for depressed patients exhibiting melancholic features. Some studies have demonstrated advantages for TCAs over SSRIs in the treatment of melancholia [29-32], and these results have found support from patient-rated assessments of antidepressant efficacy. When melancholic patients were asked to judge the extent to which previous antidepressant therapies had been effective, 38% of those who had received TCAs rated them as moderately or totally effective, compared with 22% of those who had received SSRIs [56]. However, other studies have found no significant differences in efficacy between SSRIs and TCAs in melancholic patients [33,35], or in some cases superiority of an SSRI (fluvoxamine) over a TCA (imipramine) [34,57]. In the absence of clear evidence for superior efficacy of any one class of medication, the safety and tolerability profile of the SSRIs has led to their emergence as first line treatment for melancholia. The present study found no significant differences in efficacy between duloxetine and SSRI comparators within the subgroup of melancholic patients, although we note that the studies were not powered to detect such differences. These results suggest that pharmacological treatment of patients with melancholic features should be assessed on a case-by-case basis, and emphasize that distinct class effects have yet to be demonstrated consistently within this group of patients.

Results from the present investigation must be considered in light of several limitations. Firstly, this was a post-hoc analysis of pooled clinical trial data. Although subgroup analyses of efficacy assessments stratified by melancholic status were pre-specified in each protocol, the pooling strategies and some of the analyses conducted in the present investigation were not pre-specified. Secondly, while melancholic features were evaluated as part of the MINI at study entry, specific melancholic features were not an entry requirement and randomization was not stratified by melancholic status. Furthermore, as discussed previously in some detail, the MINI is not an ideal tool for assessing the presence of melancholic features. Thirdly, the HAMD_17 _scale has only limited capability to assess improvement in melancholic symptoms. Anhedonia, one of the key features of melancholia, is only assessed indirectly as a part of question 7, while psychomotor agitation is only partially addressed by question 9. Furthermore, items such as diurnal variation in mood are not specifically measured by the HAMD_17_.

Fourthly, the group of patients receiving the recommended target dose of duloxetine (60 mg QD) could not be compared head-to-head with SSRIs, as these two studies did not feature active comparator treatment groups. Finally, although duloxetine demonstrated significant advantage over placebo on a number of efficacy measures, the individual studies were powered on the basis of the primary outcome – mean change in HAMD_17 _total score. Therefore, results from other efficacy measures should be viewed as secondary in nature. Together, these limitations necessitate that the results from the present investigation cannot be considered confirmatory. A prospectively designed clinical trial will be required to confirm the results suggested by the current set of analyses.

## Conclusions

In these analyses of pooled data, the efficacy of duloxetine in patients with melancholic features did not differ significantly from that observed in non-melancholic patients. Additional prospectively designed clinical trials evaluating duloxetine's efficacy in melancholic patients will be required to substantiate the preliminary findings described here.

## Competing interests

Drs. Mallinckrodt, Watkin, Liu, Wohlreich, and Raskin are employees of Eli Lilly and Company.

## Authors' contributions

CHM proposed the data pooling strategies, designed the statistical analyses, and participated in interpretation of data and drafting of the manuscript. MMW, JR, and JGW participated in interpretation of data and drafting of the manuscript. CL performed the statistical analyses. All authors read and approved the final manuscript.

## Pre-publication history

The pre-publication history for this paper can be accessed here:


